# Intermediate pressure-normalized principal wall strain values are associated with increased abdominal aortic aneurysmal growth rates

**DOI:** 10.3389/fcvm.2023.1232844

**Published:** 2023-08-31

**Authors:** Zachary R. Zottola, Daniel S. Kong, Ankit N. Medhekar, Lauren E. Frye, Scarlett B. Hao, Dakota W. Gonring, Adnan A. Hirad, Michael C. Stoner, Michael S. Richards, Doran S. Mix

**Affiliations:** ^1^ Division of Vascular Surgery, Department of Surgery, Cardiovascular Engineering Lab, University of Rochester Medical Center, Rochester, NY, United States; ^2^ Department of Biomedical Engineering, Rochester Institute of Technology, Rochester, NY, United States

**Keywords:** abdominal aortic aneurysms, ultrasonography, elasticity imaging techniques, aneurysmal rupture, endovascular abdominal aneurysm repair

## Abstract

**Introduction:**

Current abdominal aortic aneurysm (AAA) assessment relies on analysis of AAA diameter and growth rate. However, evidence demonstrates that AAA pathology varies among patients and morphometric analysis alone is insufficient to precisely predict individual rupture risk. Biomechanical parameters, such as pressure-normalized AAA principal wall strain ($\bar{\varepsilon_{\rho +}}$/PP, %/mmHg), can provide useful information for AAA assessment. Therefore, this study utilized a previously validated ultrasound elastography (USE) technique to correlate $\bar{\varepsilon_{\rho +}}$/PP with the current AAA assessment methods of maximal diameter and growth rate.

**Methods:**

Our USE algorithm utilizes a finite element mesh, overlaid a 2D cross-sectional view of the user-defined AAA wall, at the location of maximum diameter, to track two-dimensional, frame-to-frame displacements over a full cardiac cycle, using a custom image registration algorithm to produce $\bar{\varepsilon_{\rho +}}$/PP. This metric was compared between patients with healthy aortas and AAAs (≥3 cm) and compared between small and large AAAs (≥5 cm). AAAs were then separated into terciles based on $\bar{\varepsilon_{\rho +}}$/PP values to further assess differences in our metric across maximal diameter and prospective growth rate. Non-parametric tests of hypotheses were used to assess statistical significance as appropriate.

**Results:**

USE analysis was conducted on 129 patients, 16 healthy aortas and 113 AAAs, of which 86 were classified as small AAAs and 27 as large. Non-aneurysmal aortas showed higher $\bar{\varepsilon_{\rho +}}$/PP compared to AAAs (0.044 ± 0.015 vs. 0.034 ± 0.017%/mmHg, *p* = 0.01) indicating AAA walls to be stiffer. Small and large AAAs showed no difference in $\bar{\varepsilon_{\rho +}}$/PP. When divided into terciles based on $\bar{\varepsilon_{\rho +}}$/PP cutoffs of 0.0251 and 0.038%/mmHg, there was no difference in AAA diameter. There was a statistically significant difference in prospective growth rate between the intermediate tercile and the outer two terciles (1.46 ± 2.48 vs. 3.59 ± 3.83 vs. 1.78 ± 1.64 mm/yr, *p* = 0.014).

**Discussion:**

There was no correlation between AAA diameter and $\bar{\varepsilon_{\rho +}}$/PP, indicating biomechanical markers of AAA pathology are likely independent of diameter. AAAs in the intermediate tercile of $\bar{\varepsilon_{\rho +}}$/PP values were found to have nearly double the growth rates than the highest or lowest tercile, indicating an intermediate range of $\bar{\varepsilon_{\rho +}}$/PP values for which patients are at risk for increased AAA expansion, likely necessitating more frequent imaging follow-up.

## 1. Introduction

At an estimated 3% incidence in the general population based on random screening studies (1), abdominal aortic aneurysms (AAA) are a fatal disease process that can lead to rupture, reaching in-hospital mortality rates around 50% with estimated mortality rates around 80% when including patients who never reach the hospital (2–4). While there have been many risk factors associated with the development of aortic aneurysms such as age, gender, smoking history and family history, and many theories for the role of biological processes such as atherosclerosis and the presence of metalloproteases, the pathogenesis of aneurysm formation is not fully understood (2–4).

Based on the fatal outcomes and mortality associated with ruptured AAAs, multiple professional societies have established screening guidelines to identify and electively repair them (5–7). However, due to the unclear pathogenesis, surgeons must rely on morphometric analysis that uses ultrasound (US), magnetic resonance imaging (MRI), and computed tomography angiography (CTA) to assess aneurysmal diameter, as well as growth rates. This assessment is based on a host of previous research demonstrating aneurysmal diameter and growth rates are highly associated with risk of AAA rupture (8–10). Therefore, diameter and growth rate thresholds have been established for surgical treatment with the Society for Vascular Surgery's United States guidelines suggesting any aneurysm ≥5.5 cm in men or ≥5.0 cm in women, or with a rapid growth rate (generally 0.5 cm in 6 months or 1 cm in 1 year) meets the threshold for surgical repair (5). While these parameters identify a large proportion of patients at risk for AAA rupture, studies have demonstrated there remains a low risk of rupture in patients below these surgical thresholds, especially as it pertains to the established diameter thresholds. Additionally, many patients present having already exceeded these thresholds at the time of diagnosis without rupturing (10–12). This suggests that morphometric analysis alone is inefficient in accurately deciding intervention in all patients, as AAA formation involves both physical and biological processes and aneurysmal pathology likely differs between individual patients. In other words, by using simple diameter and growth measurements, there will be missed patients who are at risk of rupture and patients who undergo intervention unnecessarily early in their disease process. As such, additional and more precise methods of individual patient rupture risk analysis are needed.

Promising research has been undertaken into the investigation of biomechanical markers of AAA pathology, mostly in the form of tissue failure predictors, keeping in mind that tissue fails when stress on that tissue overcomes its intrinsic strength (13). Therefore, early studies began investigating the correlation between peak wall stress and AAA rupture, finding that peak wall stress was a superior marker in predicting unfavorable outcomes in AAA patients compared to maximum diameter (14–17). However, tissue failure also relies on the measurement of intrinsic tissue strength or stiffness, which varies among the circumference of the vessel wall, making peak wall stress an incomplete maker (13, 18). It should also be noted that previously, untimely specific tissue strength could only be determined by destructive testing (19), and noninvasive testing relied on predictive modeling to determine tissue strength. Thus, research in this area has turned to advanced *in-vivo* methods of analyzing more accurate biomechanical parameters with earlier studies analyzing measurements such as elastic modulus (Ep), and beta stiffness (β) (measures of tissue stiffness that also account for the stress on the material) using two-dimensional ultrasound-based echo wall tracking techniques, demonstrating the feasibility of in-vivo testing and showing late decreases in these parameters are associated with increased rupture risks in AAA patients (20–22).

While these earlier studies demonstrated Ep and β to be accurate markers of AAA pathology, earlier analysis methods have relied on linear, 2D measurements which assume an axisymmetric shape of the patient's aorta and cannot capture the biomechanical heterogeneity of the vessel. More recent work in this area has focused on similar biomechanical parameters assessed via more complex imaging methods such as 3D and 4D ultrasound analysis. For example, in 2013, Karatolios et al. demonstrated the feasibility of 3D ultrasound speckle tracking combined with Finite Element analysis to identify spatial and temporal differences in circumferential and longitudinal aortic wall strain (23). Further research has used 4D ultrasound to assess AAA pathology such as Derwich et al who used a similar 3D speckle tracking technique over a period of 24.5 months to produce a 4D analysis of circumferential aortic strain, or Cebull et al. who used a nonrigid registration algorithm via a direct deformation estimation method to demonstrate the ability of 4D ultrasound to detect Green-Lagrange strain heterogeneity in murine models (24, 25). Perhaps the most interesting is Wittek et al. who used 4D circumferential strain measurements normalized via pulse pressure to produce a value referred to as the distensibility coefficient, representing a value that can be normalized across patients in a clinical setting (25, 26).

Presented in this paper is a similar ultrasound-based method to the ones described above, the benefits of which are described in Mix et al. and Zottola et al., which uses a value called pressure-normalized AAA principal wall strain ($\bar{\varepsilon_{\rho +}}$/PP) assessed via ultrasound elastography (USE). This method considers both the stiffness of the tissue and the stress imposed on the aortic wall to assess AAA pathology, by accounting for tissue strain in both the axial and circumferential directions, as well as the circumferential stretch and compressive strain imposed on the tissue, via a custom image registration algorithm (27, 28). As elastic modulus (Ep) is defined by the slope of a stress-strain curve (stress/strain), $\bar{\varepsilon_{\rho +}}$/PP, which is a measurement of tissue strain ($\bar{\varepsilon_{\rho +}}$) normalized by the stress on the AAA tissue as represented by the patient's pulse pressure (PP), can act as the proportional inverse of Ep. Therefore, the previously described decrease in Ep (stiffness) of the aneurysmal wall that correlates with increased risk of rupture, as described in Wilson et al (21)., correlates to an increased $\bar{\varepsilon_{\rho +}}$/PP, potentially representing an increased risk for rupture. This method is conducted by imaging the axial view of the AAA at the point of maximal diameter, similar to how standard screening studies are currently conducted, providing a clinically applicable and easily integrated method for measuring aneurysmal strain and vessel wall properties.

We hypothesize our analysis method could be an additional non-invasive tool in AAA screening and preoperative assessment. Therefore, this study aimed to correlate this method of AAA assessment with the existing predictors of AAA rupture by comparing $\bar{\varepsilon_{\rho +}}$/PP to maximal AAA diameter and growth rate in a large patient cohort.

## 2. Materials and methods

### 2.1. Study design

This is a retrospective review of prospectively collected, single-institutional data of patients with both infrarenal and suprarenal AAAs. Patients were imaged using ultrasound (US) and analyzed using our USE protocol over a maximum period of 5 years. $\bar{\varepsilon_{\rho +}}$/PP was calculated and correlated to AAA diameter, prospective growth rate, retrospective growth rate and time to surgical repair. Additional demographic and imaging information was taken from the patient's electronic medical record. The present study and informed consent process were reviewed and approved by the IRB office of the Research Subjects Review Board of the University of Rochester.

### 2.2. Patient recruitment

Patients were identified and enrolled through the vascular surgery department of the University of Rochester Medical Center from July 2015 to August 2016. Patients were recruited based on the need for a standard US screening exam for either a suspected, new, or existing non-repaired aneurysm with an abdominal component (infrarenal or suprarenal extending through the abdomen). Exclusion criteria included patients who were unable to provide adequate consent, such as patients under eighteen and special populations such as prisoners and pregnancy. All patients enrolled gave written informed consent prior to imaging.

### 2.3. Ultrasound scan protocol

Our USE protocol has been published in detail by Mix et al. and briefly reviewed here (27, 28). All patients were imaged in our outpatient clinic concurrently with their scheduled screening US exams. USE imaging was conducted using either the Ultrasonix Sonix-Touch US System (BK Medical, Burlington, MA) or the Ultrasonix Sonix-Tablet US System along with an Ultrasonix C7-3/50 convex transducer. B-mode US at a frequency of 3 or 5 MHz was used to capture the abdominal aorta, in transverse orientation, at the point of maximal diameter determined by the sonographer. Sector and depth settings were set to maximize the recorded frame rate. A 10-second breath hold was performed to prevent physiologic displacement of the aorta. Radiofrequency (RF) data was recorded and analyzed by our image analysis algorithm. Blood pressure measurements were taken using a manual sphygmomanometer and used to calculate pulse pressure, which was later used to calculate $\bar{\varepsilon_{\rho +}}$/PP.

### 2.4. Ultrasound analysis protocol

The presented USE analysis algorithm has been published and validated in previous studies (27–30). Our MATLAB (2019b, Natick, Massachusetts, MathWorks Inc., RRID: SCR_001622) algorithm uses a finite element mesh, overlaid a 2D cross-section of the aorta at the location of maximum diameter, to track the two-dimensional, frame-to-frame displacements of each element over a full cardiac cycle, using a custom image registration algorithm. The total accumulated displacement map and the brachial pulse pressure are used to quantify the spatial variations in the pressure-normalized principal AAA wall strain ($\bar{\varepsilon_{\rho +}}$/PP) and subsequently, the average value within the vessel wall (27, 28).

**Figure 1:**
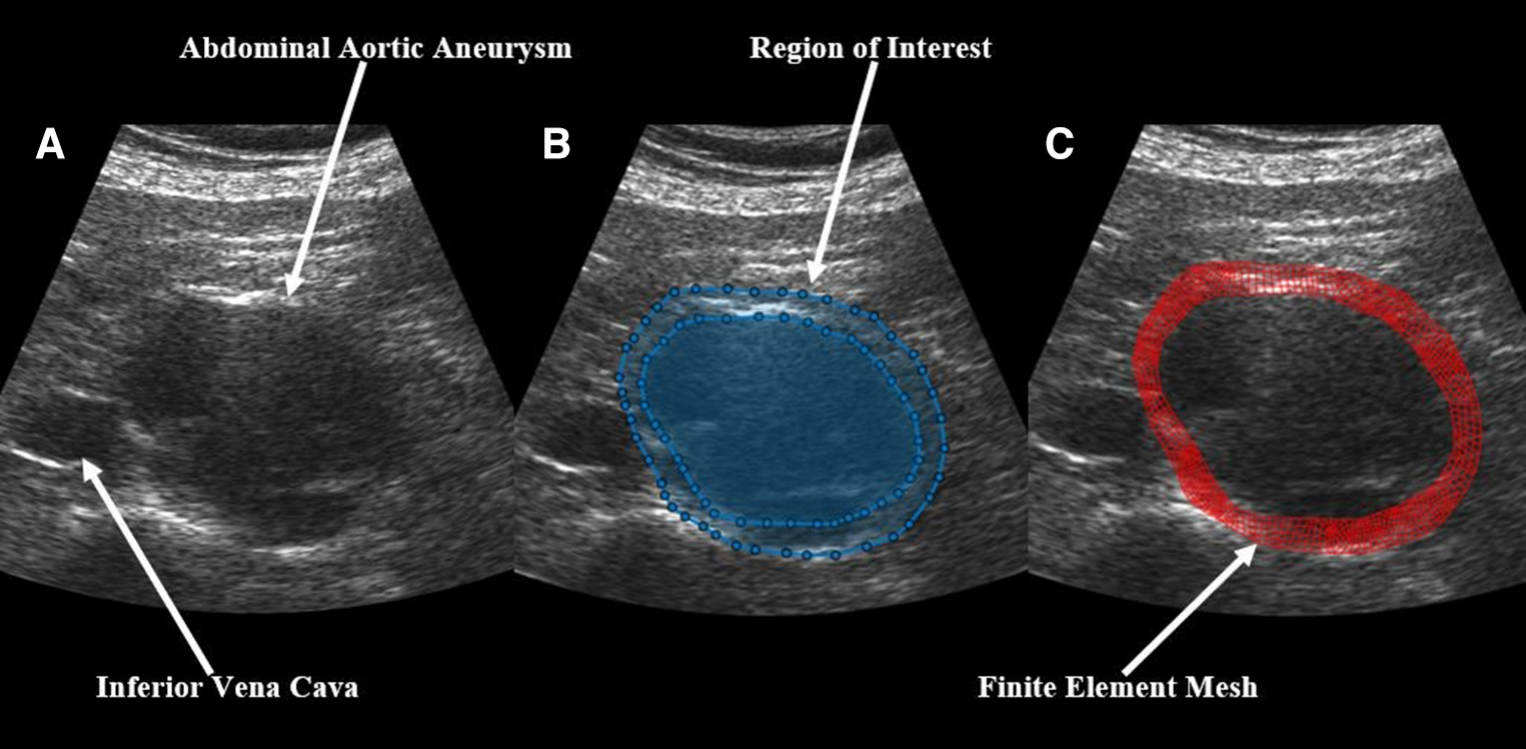
Demonstration of the construction of a finite element mesh over a patient's abdominal aortic aneurysm showing (**A**) a baseline B-mode ultrasound image, (**B**) the same ultrasound image with overlaid, user-identified regions of interest, and (**C**) the final finite element mesh construction over the patient's aorta.

**Figure 2:**
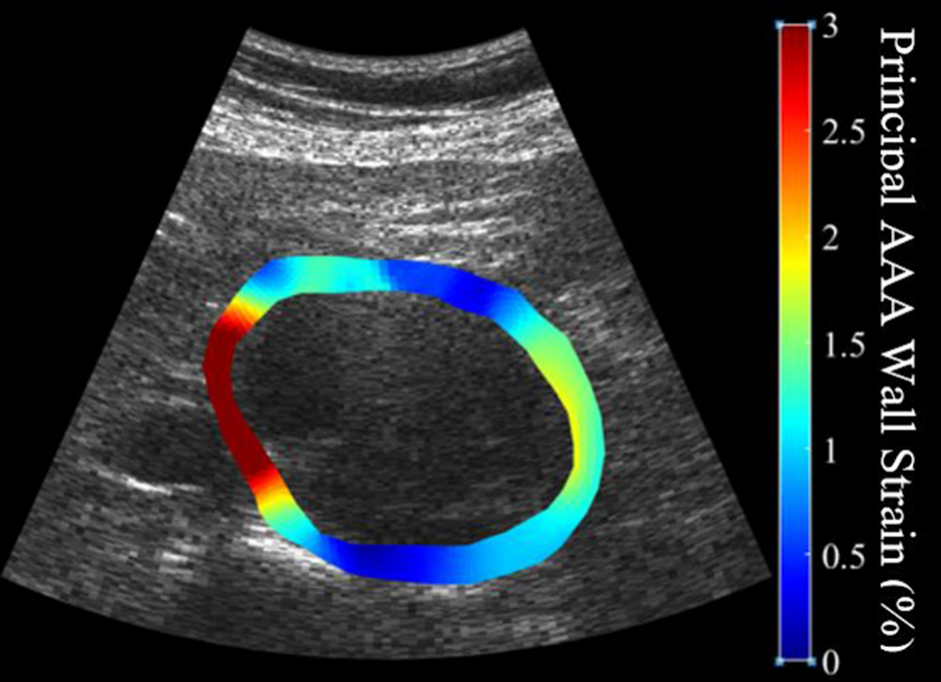
Illustration of the heterogeneity of strain measurements throughout the circumference of a patient's abdominal aortic aneurysm demonstrating high strains (softer tissue) in red and low strains (stiffer tissue) in blue.

In brief, US RF data was converted to B-mode cine loops with the interpreter isolating the frames of one full cardiac cycle. The interpreter then marked the borders that would make up the region of interest for finite element mesh construction, with the first border representing the lumen of the vessel and the second border representing the outer wall of the vessel (Figure 1). Thrombus was included in the region selection as thrombus has been shown to impact strain and modulus calculations (13, 31). The displacement of each node within the constructed finite element mesh was then tracked from frame to frame to calculate the strain of each element within the mesh. The average strain of all elements within the mesh were then calculated to give the mean principal strain for a given frame. The accumulated spatial mean principal strains for each frame were then graphed, and the maximum mean principal strain ($\bar{\varepsilon_{\rho +}}$) was determined. From here, parametric imaging was used to show principal regional strain within the entire aorta in the frame of $\bar{\varepsilon_{\rho +}}$ (Figure 2). $\bar{\varepsilon_{\rho +}}$ was then divided by pulse pressure taken at the time of the scan to produce $\bar{\varepsilon_{\rho +}}$/PP (27, 28). Additionally, a minimum regional cross-correlation (MRCC) value was calculated for all analyses, which is a value that assesses how well the image processing can maintain an adequate lock on the region of interest and is highly reliant on US image quality. A MRCC below zero was felt to represent an inadequate assessment of the patient's aorta and patients with a sub-zero value were excluded.

A random subset of the study population was analyzed twice, each by a study team member. An intraclass correlation coefficient (ICC), using a two-way random effects model to assess for absolute agreement, and a Bland-Altman plot were created to ensure adequate correlation between the $\bar{\varepsilon_{\rho +}}$/PP values of the two interpreters. As values were not normally distributed, $\bar{\varepsilon_{\rho +}}$/PP values were log-transformed before analysis to satisfy the normality assumptions of the ICC analysis and Bland-Altman plot (32, 33).

### 2.5. Outcomes and clinical patient variables

Primary outcomes included AAA diameter and prospective growth rate. Secondary outcomes included retrospective growth rate, incidence of surgery and time to intervention. All AAA diameters were recorded from the patient's first screening US exam within the study. Patient prospective growth rates were calculated for all patients in the AAA group with at least one follow-up, using either their most recent follow-up US scan or their pre-surgical US scan. Retrospective growth rates were calculated using any previously available US scan. All AAA sizing was determined by a member of the study team using the maximal diameter of the transverse aorta in an axial view. Aneurysm diameter was measured using ultrasound with the probe orientated in the transverse position relative to the tortuosity of the abdominal aorta. The diameter measurement was defined as the maximum, outer wall to outer wall, end diastolic measurement of the aorta in this 2D axial view. This was the same point used to capture the B-mode cine loop to be used in strain algorithm calculations. Patient clinical variables including demographic data (age, sex, race, smoking status), past medical history (hypertension, type II diabetes mellitus, chronic kidney disease, chronic obstructive pulmonary disease, cancer, atrial fibrillation), medication data (anticoagulation, angiotensin converting enzyme inhibitors, statins, beta blockers, tacrolimus, cyclosporine), the incidence of surgery, and time to the intervention were obtained through patient electronic medical records.

### 2.6. Statistical analysis

Patients were divided into a normal (healthy) aortic group and a AAA group based on a AAA diameter of ≥3 cm. Clinical variables were compared between groups using Fisher's exact tests and Mann–Whitney *U* tests for categorical and continuous variables, respectively. $\bar{\varepsilon_{\rho +}}$/PP values were compared between groups using a Mann–Whitney *U* test. Patients in the AAA group were then divided into large and small aneurysms based on a AAA diameter of ≥5 cm. Clinical variables and $\bar{\varepsilon_{\rho +}}$/PP values were then compared between groups as described above. Linear associations between $\bar{\varepsilon_{\rho +}}$/PP and AAA diameter, as well as $\bar{\varepsilon_{\rho +}}$/PP and prospective and retrospective growth rates were assessed using Spearman's rank correlations.

**Figure 3:**
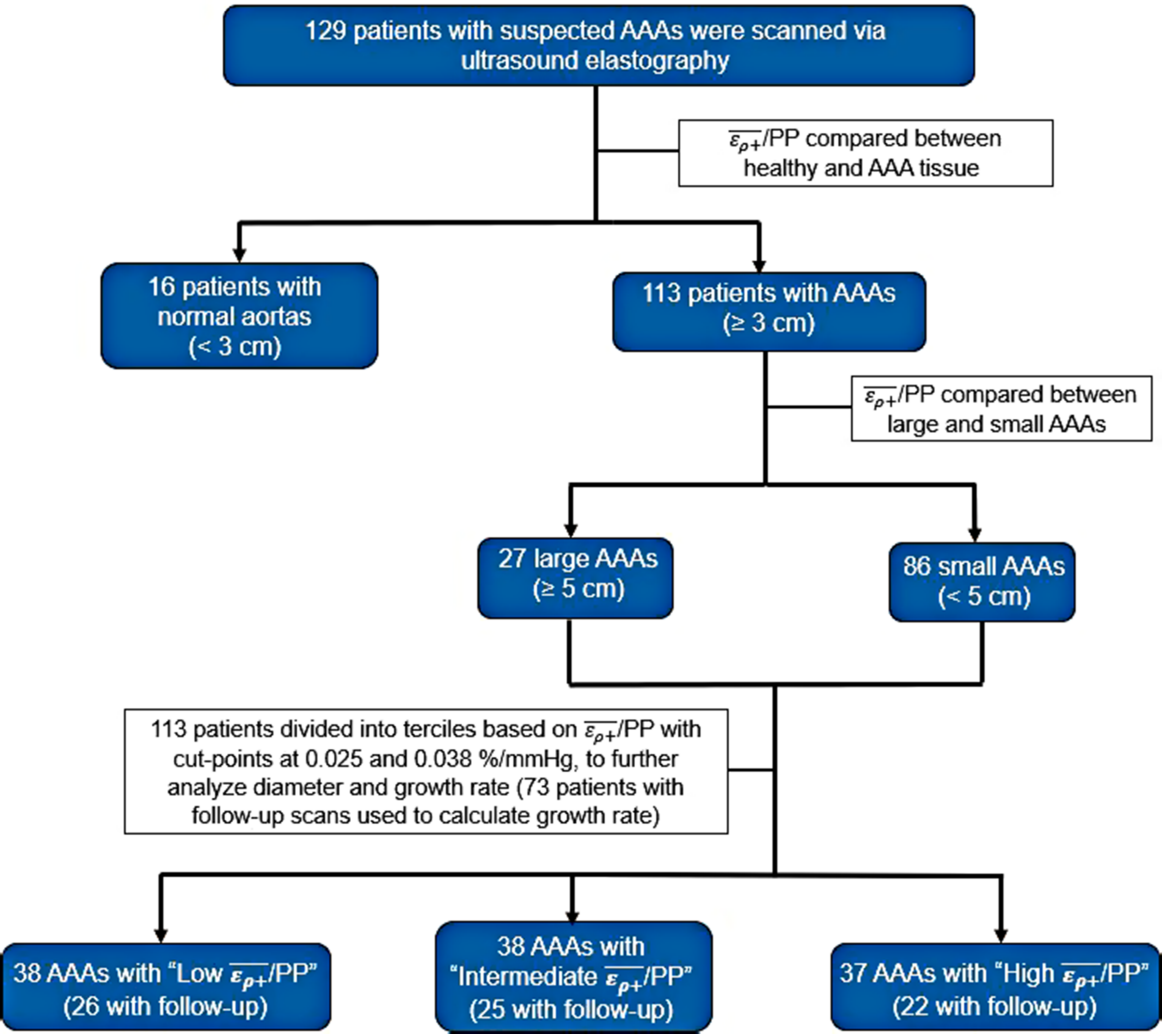
Flow map demonstrating the grouping of patients into various AAA and $\bar{\varepsilon_{\rho +}}$/PP cohorts for analysis.

Patients in the AAA group were then divided into terciles based on the $\bar{\varepsilon_{\rho +}}$/PP values of the study population. Differences in AAA diameter, prospective growth rate, retrospective growth rate, the incidence of surgery, and time to repair between the terciles were assessed via non-parametric Kruskal–Wallis tests. Significant variables were then compared between terciles using a Dunn's pairwise multiple comparison test to further elucidate difference among groups (34). A flow-map illustrating creation of patient cohorts is displayed in Figure 3. Statistical significance was considered using a two-sided *p*-value of ≤0.05 for all analyses. Statistical analysis was performed using STATA 17.0 (STATAcorp, College Station, TX, RRID: SCR_012763).

## 3. Results

**Figure 4:**
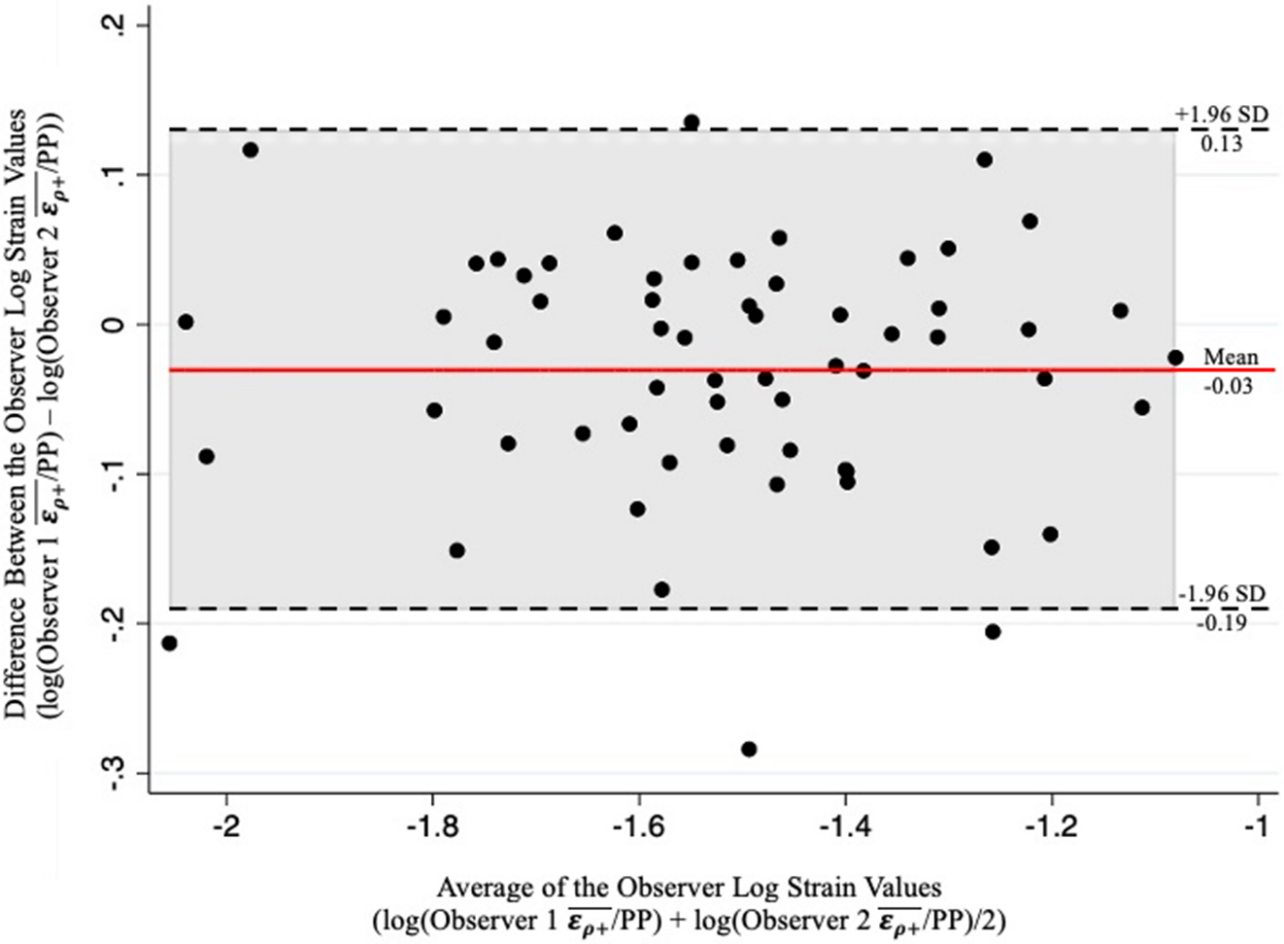
Bland-Altman plot of the results from two-observer analysis demonstrating adequate inter-operator correlation of the log-transformed $\bar{\varepsilon_{\rho +}}$/PP values as evidenced by a minimal number of obeservations outside the upper and lower limits.

A total of 134 patients with infrarenal AAAs, suprarenal AAAs and non-aneurysmal aortas were included in this study. Of the 134 patients, 4 patients with infrarenal AAAs and 1 patient with a non-aneurysmal aorta were excluded from the study based on MRCC values less than zero. A total of 50 patients were lost to follow up, meaning a second USE examination was never conducted. Of the 79 patients with follow up, 6 exhibited normal aortas and 73 were AAAs (71 infrarenal and 2 suprarenal). The mean follow-up time was 2.4 years. Previous US scans were available for 101 patients with a mean retrospective time of 1 year. Analysis of the inter-operator differences in the assessment of $\bar{\varepsilon_{\rho +}}$/PP of 61 patients showed adequate inter-operator absolute agreement based on a ICC of 0.93 (*p* < 0.001). An accompanying Bland-Altman plot is represented in Figure 4 showing adequate agreement with only 4 observations outside the accepted upper and lower limits (Figure 4).

**Figure 5:**
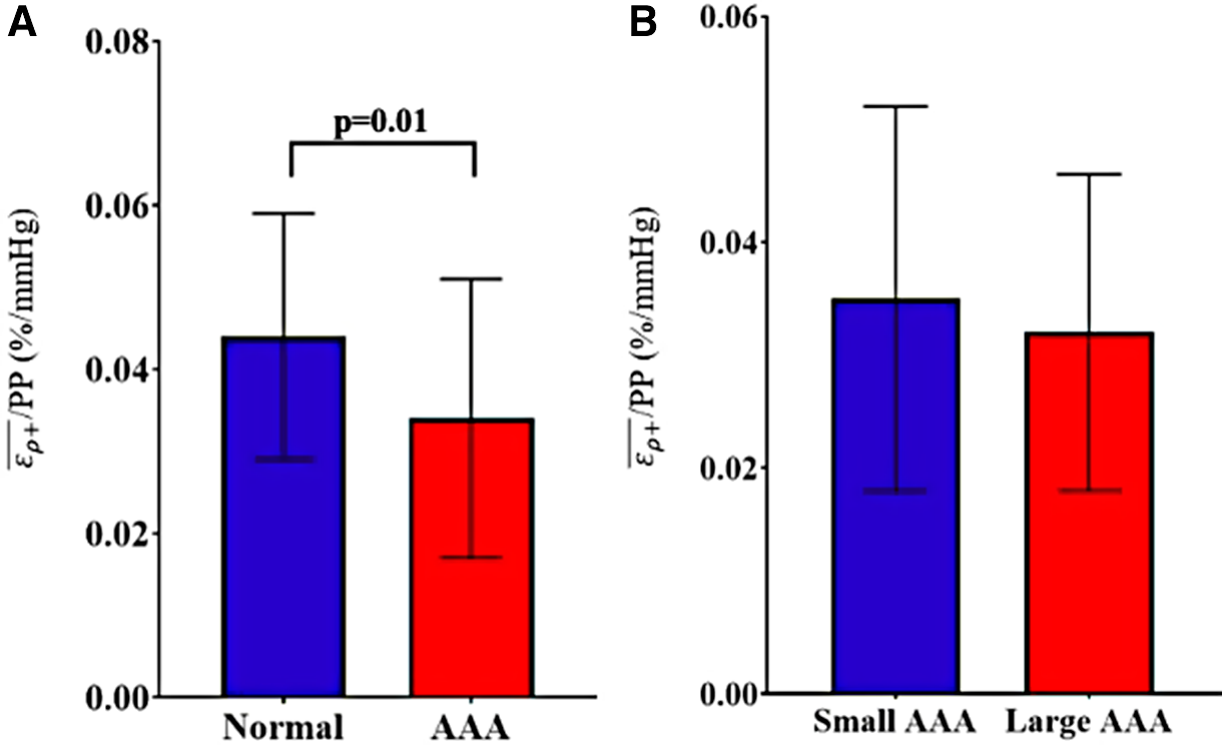
Comparison of $\bar{\varepsilon_{\rho +}}$/PP between (**A**) normal (healthy) aortas versus AAAs and (**B**) small AAAs versus large AAAs, presented as mean ± standard deviation.

Grouped based on a diameter of ≥3 cm, the study sample consisted of 16 non-aneurysmal aortas and 113 AAAs (3 suprarenal and 110 infrarenal). There were no significant differences in any clinical variables between the normal aortas and AAAs except for active smoking status (0% vs. 27.3%, *p* = 0.02) (Table 1). The normal aortic group demonstrated statistically significant higher $\bar{\varepsilon_{\rho +}}$/PP compared to the AAA group (Mean ± Std. Dev.: 0.044 ± 0.015 vs. 0.034 ± 0.017%/mmHg, *p* = 0.01) (Figure 5). Based on a diameter of ≥5 cm, the sample consisted of 86 small AAAs and 27 large AAAs. There were no significant differences in any clinical variables between groups (Table 1). There were no statistically significant differences in mean $\bar{\varepsilon_{\rho +}}$/PP (0.035 ± 0.017 vs. 0.032 ± 0.014%/mmHg, *p* = 0.58) (Figure 5). Spearman's rank analysis demonstrated no significant correlation between $\bar{\varepsilon_{\rho +}}$/PP and AAA diameter (*r_s_*_ _= −0.03, *p* = 0.72), prospective growth rate (*r_s_*_ _= 0.12, *p* = 0.33), or retrospective growth rate (*r_s_*_ _= 0.09, *p* = 0.41).

**Table 1:** Comparison of patient demographics between normal aortas versus AAAs, and small vs large AAAs, including comparisons of pressure-normalized principal AAA wall strain ($\bar{\varepsilon_{\rho +}}$/PP).

	Normal VS AAA			Small AAA VS Large AAA		
	Normal (*n* = 16)	AAA (*n* = 113)	*p*-value	Small (*n* = 86)	Large (*n* = 27)	*p*-value
Age, years	72.1 ± 7.5	72.5 ± 11.6	0.34	71.9 ± 12.3	74.7 ± 9.3	0.33
Race, Caucasian	16 (100%)	108 (95.6%)	1.00	83 (96.5%)	25 (92.6%)	0.34
Sex, Female	4 (25%)	20 (17.7%)	0.50	16 (18.6%)	4 (14.8%)	0.78
Active Smoker	0 (0%)	27 (27.3%)	**0** **.** **02**	22 (29.0%)	5 (21.7%)	0.60
Hypertension	11 (68.8%)	59 (52.2%)	0.29	46 (53.5%)	13 (48.2%)	0.66
DM II	3 (18.8%)	17 (15.0%)	0.71	15 (17.4%)	2 (7.4%)	0.35
Statin	13 (81.3%)	83 (73.5%)	0.76	64 (74.4%)	19 (70.4%)	0.80
ACE Inhibitor	7 (43.8%)	54 (47.8%)	0.80	41 (47.7%)	13 (48.2%)	1.00
Beta Blocker	7 (43.8%)	53 (46.9%)	1.00	41 (47.7%)	12 (44.4%)	0.83
Atrial Fibrillation	0 (0%)	20 (17.7%)	0.13	15 (17.4%)	5 (18.5%)	1.00
Anticoagulation	0 (0%)	20 (17.7%)	0.13	13 (15.1%)	7 (25.9%)	0.25
CKD	0 (0%)	13 (11.5%)	0.37	9 (10.5%)	4 (14.8)	0.51
COPD	1 (6.3%)	24 (21.2%)	0.31	19 (22.1%)	5 (18.5%)	0.79
Tacrolimus	0 (0%)	3 (2.7%)	1.00	3 (3.5%)	0 (0%)	1.00
Cyclosporine	0 (0%)	1 (0.9%)	1.00	1 (1.2%)	0 (0%)	1.00
Neoplasm	5 (31.3%)	33 (29.2%)	1.00	29 (33.7%%)	4 (14.8%)	0.09
$\bar{\varepsilon_{\rho +}}$/PP, %/Mmhg	0.044 ± 0.015	0.034 ± 0.017	**0**.**01**	0.035 ± 0.017	0.032 ± 0.014	0.58

Continuous variables are expressed as average ± standard deviation. Categorical variables are expressed as number (frequency). AAA, abdominal aortic aneurysm; DM II, diabetes mellitus type II; ACE, angiotensin converting enzyme; CKD, chronic kidney disease; COPD, chronic obstructive pulmonary disease.

Bold values denote statistical significance (*p* < 0.05).

**Figure 6:**
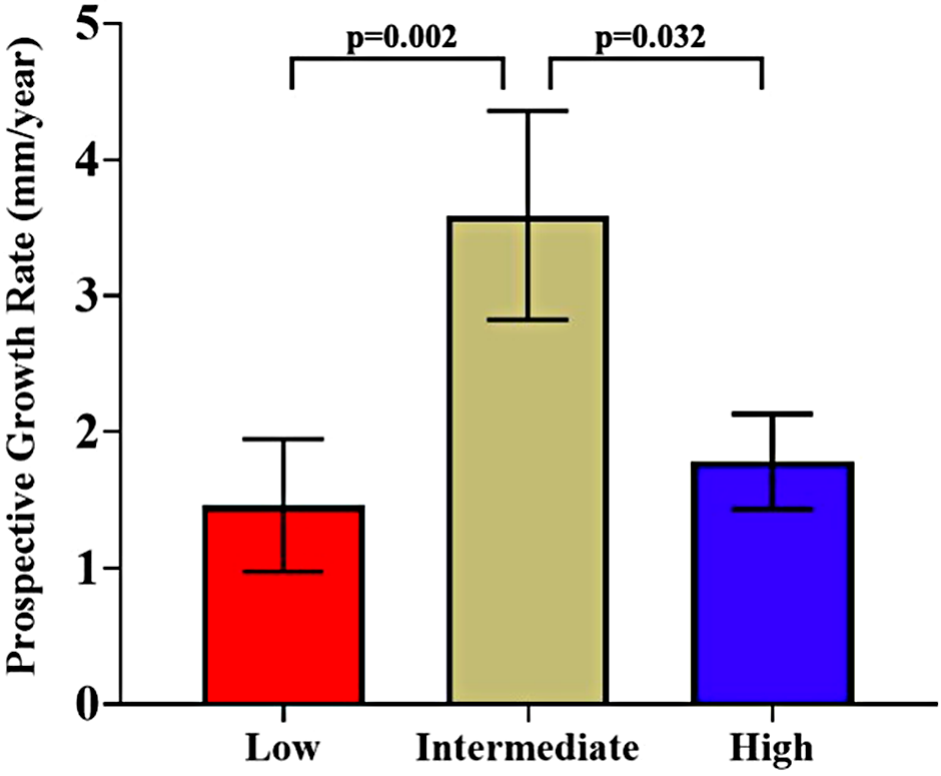
Comparison of prospective growth rates between the three $\bar{\varepsilon_{\rho +}}$/PP terciles (cut-offs at 0.0251%/mmHg and 0.038%/mmHg), presented as mean ± standard error.

After dividing patients into terciles based on $\bar{\varepsilon_{\rho +}}$/PP cutoff values of 0.0251 and 0.038%/mmHg, there was no statistically significant difference in AAA diameter, retrospective growth rate, incidence of surgery or time to surgical repair. There was a statistically significant difference in prospective growth rate (1.46 ± 2.48 vs. 3.59 ± 3.83 vs. 1.78 ± 1.64 mm/yr, *p* = 0.014) (Table 2). Upon comparison between the intermediate tercile and the two outer terciles it was confirmed that the intermediate tercile demonstrated statistically significant faster growth rates compared to both the lower (1.46 ± 2.48 vs. 3.59 ± 3.83 mm/yr, *p* = 0.002) and upper terciles (3.59 ± 3.83 vs. 1.78 ± 1.64 mm/yr, *p* = 0.032) (Table 3 and Figure 6).

**Table 2:** Comparison of prevalent AAA outcomes with all AAA patients separated into three groups by $\bar{\varepsilon_{\rho +}}$/PP terciles.

	Low ($\bar{\varepsilon_{\rho +}}$/PP ≤0.0251%/mmHg) (*n* = 38)	Mid (0.0251<$\bar{\varepsilon_{\rho +}}$/PP <0.0380%/mmHg) (*n* = 38)	High ($\bar{\varepsilon_{\rho +}}$/PP ≥0.0380%/mmHg) (*n* = 37)	*p*-value
AAA Diameter (cm)	4.43 ± 0.88	4.58 ± 0.91	4.33 ± 0.98	0.310
Prospective Growth Rate (mm/yr)	1.46 ± 2.48	3.59 ± 3.83	1.78 ± 1.64	**0** **.** **014**
Retrospective Growth Rate (mm/yr)	2.64 ± 8.01	2.76 ± 5.60	2.61 ± 3.08	0.740
Surgery	15 (39.5%)	23 (60.5%)	19 (51.4%)	0.190
Time To Repair (Yrs)	1.06 ± 1.24	1.14 ± 1.13	1.11 ± 1.33	0.790

Continuous variables are expressed as average ± standard deviation. Categorical variables are expressed as number (frequency). AAA, abdominal aortic aneurysm, cm, centimeters; mm, millimeters; yr, years.

Bold values denote statistical significance (*p* < 0.05).

**Table 3:** Three-legged analysis of prospective AAA growth rate comparing individual $\bar{\varepsilon_{\rho +}}$/PP terciles.

	Low VS Intermediate			Low VS High			Intermediate VS High		
	Low (*n* = 26)	Intermediate (*n* = 25)	*p*-value	Low (*n* = 26)	High (*n* = 22)	*p*-value	Intermediate (*n* = 25)	High (*n* = 22)	*p*-value
Prospective Growth Rate (mm/yr)	1.46 ± 2.48	3.59 ± 3.83	**0.002**	1.46 ± 2.48	1.78 ± 1.64	0.178	3.59 ± 3.83	1.78 ± 1.64	**0.032**

Variables are expressed as average ± standard deviation. Mm, millimeters; yr, years.

Bold values denote statistical significance (*p* < 0.05).

## 4. Discussion

It is generally understood that AAA pathology results from a loss of elastin content relative to normal aortic tissue, resulting in the ballooning of the abdominal aorta as the tissue becomes unable to withstand the force of physiologic pulsation. Additional earlier research demonstrates AAA tissue to be stiffer than normal aortic tissue due to this reduced elastin content and reliance of the AAA on the remaining adventitial layer to provide structural integrity and strength, which appears counter-intuitive as stiffening of the aortic tissue should prevent pathologic ballooning of the aneurysm (13, 35–37). In a 2019 histopathology study, Niestrawska et al. describe the histologic progression of AAAs, demonstrating there is a substantial loss of the elastic lamina early in the disease process with a gradual thickening of the adventitial layer accompanied by an increase in anisotropy of this layer (38). This nuance is what seems to account for the early ballooning of the aorta and formation of the AAA, which is then accompanied by a gradual stiffening of the aortic tissue over time. This stiffening of AAAs compared to normal aortic tissue has previously been proven by *in-vivo* analysis methods using magnetic-resonance elastography (MRE) and 3D US (39, 40). Our findings here coincide with the existing literature, demonstrating that AAAs exhibits reduced $\bar{\varepsilon_{\rho +}}$/PP values, which is analogous to an increased stiffness (i.e., Ep) of the tissue and concur with the earlier and later histopathologic studies.

On the subject of AAA diameter, the presented analysis demonstrated no linear correlation between $\bar{\varepsilon_{\rho +}}$/PP and the initial presenting AAA diameter at the time of the scan. Similarly, there was no difference in $\bar{\varepsilon_{\rho +}}$/PP upon comparison of small and large AAAs. Pathophysiologic theory would suggest there should be a continuation of the same aneurysmal remodeling process that is present between normal aortic tissue and AAAs as they grow larger, in that there would be further loss of elastin content and a more substantial growth of the adventitial layer. Therefore, larger aneurysms would become stiffer and show decreased $\bar{\varepsilon_{\rho +}}$/PP values. However, this does not seem to be the case. A host of other studies have similarly investigated this aspect demonstrating controversial findings. Wilson et al., in their study of 60 AAAs analyzed using another US based method, found a positive linear correlation between maximal aortic diameter and elastic modulus (Ep) (*r_s_*_ _= 0.22, *p* < 0.05) demonstrating that larger aneurysms may be stiffer than smaller ones (41). However, it has been argued that Ep is a pressure-dependent value and that beta stiffness (β) may be a more accurate biomechanical measurement of arterial vessels, to which they found no significant correlation with AAA diameter (41). Similarly, another US-based study by Long et al. of 56 AAAs found evidence of increasing compliance using simplified measurements of aortic diameter changes, but found no significant linear correlation between AAA diameter and Ep or β, nor did they find any significant difference in these values when patients were stratified by AAA diameters of ≥45 mm (42). Other, more recent studies using more advanced imaging methods such as time-resolved 3D US and MRE show similar contradicting results, with the 3D US study showing a significant difference in aortic stiffness between 35 small (30–39 mm) AAAs and 52 large (≥50 mm) AAAs, as well as a significant linear correlation between stiffness and AAA diameter (*ρ *= 0.33, *p* = 0.007) (39). However, MRE analysis of 72 AAAs found no correlation between AAA stiffness and diameter (43). Similarly, Derwich et al. and Wittek et al. demonstrated no correlation between aortic stiffness and diameter measurements in 18 and 64 AAAs respectively, using 4D ultrasound to analyze patients (24, 26).

It is difficult to interpret these controversial results. In one manner, the contradicting information and differences among studies could be evidence that biomechanical parameters of AAAs are, in fact, unrelated to AAA diameter, providing evidence for the theory that morphometric analysis of AAAs alone is an inadequate measure of screening and surgical planning. Adding strength to this argument is the finding in Wilson et al. that despite a positive correlation between diameter and Ep, AAAs of similar sizes demonstrated up to 10-fold variations in Ep, highlighting the unreliability of this finding (41). Additionally, the studies that found statistically significant correlations between stiffness and diameter produce sub-0.35 Spearman's rank coefficients, which indicates a weak correlation. Similarly, many of the more recent studies using more advanced imaging techniques have found no correlation between biomechanical parameters of AAA tissue and diameter. However, these findings are in the setting of studies that use different imaging methods, making direct comparisons difficult and making any definitive interpretation impossible. Our analysis found no correlation between diameter and $\bar{\varepsilon_{\rho +}}$/PP. This is in the face of a reduced large AAA group consisting of 27 AAAs ≥5 cm, presenting a major limitation. However, it seems to appear that the presented collection of data and previous studies presents evidence that diameter is unrelated to biomechanical parameters and is unable to precisely predict individual patient rupture risk alone, indicating stronger analysis methods for the assessment of AAAs are needed.

In contrast to AAA diameter, few previous studies have examined measures of AAA wall biomechanics related to AAA growth rates. Of the studies that have examined values of Ep, β, and other measures of aortic stiffness, none have found any significant correlation between AAA wall biomechanics and prospective growth rates (39, 41, 44). The presented analysis also failed to find any statistically significant linear correlation between prospective growth rate and $\bar{\varepsilon_{\rho +}}$/PP. However, in the presented study, AAAs were also separated by terciles, based on the authors’ theory that AAAs with very low $\bar{\varepsilon_{\rho +}}$/PP, indicating very stiff aneurysms, likely have a high collagen crosslinking that would allow for adequate strength to resist deformation during pulsation and further expansion, and that AAAs with very high $\bar{\varepsilon_{\rho +}}$/PP, indicating soft aneurysms, likely have near physiologic levels of elastin content that would allow the aneurysmal wall to incur high strains during pulsation, but still return to a diastolic baseline diameter without baseline expansion. Therefore, those AAAs that fall into the intermediate category would have insufficient elastin content to maintain a baseline diastolic diameter in the face of pulsatility and poor collagen crosslinking to resist deformation, leading to an overwhelming of the collagen remodeling process and expansion of the aneurysm until rupture. The presented analysis then demonstrates that the intermediate $\bar{\varepsilon_{\rho +}}$/PP tercile of AAAs exhibit statistically significant higher prospective growth rates while starting at a statistically identical average AAA diameter baseline compared to the upper and lower terciles. Therefore, there appears to be a range of $\bar{\varepsilon_{\rho +}}$/PP values, around 0.025%–0.035%/mmHg, which represent a risk of increased AAA growth.

Interestingly the author's theory and *in-vivo* biomechanical findings appear to be supported by previous histopathological data. In Niestrawska et al. the authors intricately describe their findings related to the progression of AAA disease in three stages (38). Per their findings, the first stage of aneurysmal degeneration is the previously described loss of the elastic lamina, which is accompanied by a small increase in intimal thickness, leading to the formation of the aneurysm. Within stage two, the adventitial layer begins to thicken and starts forming a “neo-adventitia” as a result of disrupted adipocyte and inflammatory cell infiltration. Within stage two, the authors also describe a bursting of the intimal layer resulting in an increased compliance of the of the AAA tissue. In the presented study, the authors believe that the high $\bar{\varepsilon_{\rho +}}$/PP tercile likely correlates to stage two of the process described by Niestrawska et al., where high $\bar{\varepsilon_{\rho +}}$/PP, due to a compliant aortic wall, represents the continued loss of the elastic laminal layer accompanied by the bursting of the intimal layer resulting in high AAA wall compliance. Entering stage three, the neo-adventitial layer thickens further, resulting in a stiffening of the AAA wall, which would correspond to the low $\bar{\varepsilon_{\rho +}}$/PP tercile and a stabilizing of the aneurysm. As such the intermediate $\bar{\varepsilon_{\rho +}}$/PP tercile likely corresponds to a point between stage two and stage three, where the neo-adventitial layer is being formed but remains inadequate, the elastic lamina is nearly absent, and the intimal layer has ruptured, resulting in no substantial structural integrity of the aneurysm and an increased growth rate. Niestrawska et al. continue to describe a second type of stage three (38). While some aneurysms remodel “safely” with histologic evidence suggesting regression of inflammatory cells and disrupted adipocytes, some stage three AAAs remodel to a vulnerable state wherein the neo-adventitial layer has formed, but there is histologic evidence for the persistence of inflammatory cells and disrupted adipocytes. The authors believe that this type of vulnerable remodeling is likely also represented by the intermediate $\bar{\varepsilon_{\rho +}}$/PP tercile, as an aneurysm with continued inflammation would likely tend to have an increased growth rate without a final formalization of the neo-adventitial layer. Further direct histologic evidence via USE scanning of patients compared to tissue analysis of samples from scanned patients would be needed to adequately prove this theory, but the parallels are thought-provoking, nonetheless.

Despite the difference in AAA prospective growth rate between the analyzed terciles, there was no difference in retrospective growth rate, which was analyzed to assess if strain was related to previous growth, or simply a predictor of future growth. There was no linear correlation found between retrospective growth rate and $\bar{\varepsilon_{\rho +}}$/PP, nor was there any difference found when separating patients into terciles. This represents an important finding, namely that aneurysmal expansion is likely non-linear, in that previous aneurysmal growth does not necessarily predict future growth and that $\bar{\varepsilon_{\rho +}}$/PP measurements may provide a method to more accurately predict AAA expansion or the cessation of expansion in growing aneurysms.

Lastly, there was no difference in the incidence of surgery or time to surgical intervention among the terciles. This is likely due to the average prospective growth rate of the intermediate tercile equating to 3.6 mm/yr, which is drastically under the required 10 mm/yr of growth to warrant intervention based on current guidelines. Our findings could provide evidence that despite the increased growth rate in this cohort, it may be insignificant from a clinical standpoint. However, the overwhelming significance in the face of the small sample size presented here, and the previously established association of AAA growth and rupture risk (8, 9), provide an argument that patients who fall in this intermediate $\bar{\varepsilon_{\rho +}}$/PP range would likely benefit from more frequent follow up in their pre-surgical screening process.

The presented study is not without limitations. First off are the limitations inherent to the USE imaging protocol and analysis that have been previously described (27, 28). As the analysis utilizes US, it relies heavily on image quality, which can be hindered by patient body habitus, movement artifacts during the scan, inability to conduct a breath hold during the scan, and skill variation in sonographers. We mitigated errors in the scan using a minimal regional cross correlation value, in which patients with values below zero were excluded based on poor image tracking during the algorithm, and the use of two certified image interpreters that demonstrated good inter-observer agreement. It is also well-known that $\bar{\varepsilon_{\rho +}}$/PP is heterogeneous along the vessel wall and thus, analysis of one axial view of the aorta may be insufficient to accurately calculate $\bar{\varepsilon_{\rho +}}$/PP of the entire aneurysm. Multiple cross-sections, or more advanced imaging methods such as 3D-ultrasound, can be used in future analyses to mitigate this. As it relates to the interpretation of this study population, the investigation was limited by a low number of control patients, only including 16 non-aneurysmal aortas. Similarly, the study comprised only 27 AAAs in the large AAA cohort. This study also suffered from significant loss to follow up with 38.8% of patients never receiving a second follow-up USE scan. As such, there may have been significant differences that this study was too underpowered to detect. Similarly, it should be noted that following the Dunn's pairwise multiple comparison test in the statistical methods, no correction was made to account for the family-wise error rate. This was done as the authors' goal for this study was to be of an exploratory nature and to spark further research into the association of strain measurements and growth rate. As such, the authors worried about the consequence of reducing statistical power and masking a true association. Additionally, as this was a pre-planned statistical method based on the authors' hypothesis regarding the pathology of aneurysms, a correction is not necessary. Lastly, the findings of Niestrawska et al., provide a potential histological basis for the presented strain findings, adding to the argument against correcting the presented *p*-values as previous evidence supports the statistical findings (38, 45, 46).

## 5. Conclusion

In the presented study, using a validated USE imaging method, it was demonstrated that patients with AAAs have significantly decreased $\bar{\varepsilon_{\rho +}}$/PP values indicating much stiffer aortas than patients with non-aneurysmal aortas. There was no correlation between AAA diameter and $\bar{\varepsilon_{\rho +}}$/PP, which provides evidence to the theory that biomechanical markers of AAA pathology are independent of diameter measurements and that AAA diameter thresholds alone are insufficient to precisely assess the need for intervention and rupture risk in individual patients. While there was no linear correlation between $\bar{\varepsilon_{\rho +}}$/PP and prospective growth rates, patients that fell in the intermediate tercile of the study population were found to have statistically significant greater prospective growth rates than patients in the highest or lowest tercile regardless of retrospective growth. This indicates that there is likely an intermediate range of $\bar{\varepsilon_{\rho +}}$/PP values for which patients are at risk for increased AAA growth, potentially necessitating more frequent follow-up.

## Data Availability

The datasets presented in this article are not made publicly available for the protection of patient privacy and HIPPA related information. Requests to access the dataset should be directed to the corresponding author, DM at dmix@urmc.rochester.edu.
